# Correction: Tian et al. The Underlying Role of the Glymphatic System and Meningeal Lymphatic Vessels in Cerebral Small Vessel Disease. *Biomolecules* 2022, *12*, 748

**DOI:** 10.3390/biom13040705

**Published:** 2023-04-21

**Authors:** Yu Tian, Mengxi Zhao, Yiyi Chen, Mo Yang, Yilong Wang

**Affiliations:** 1 Department of Neurology, Beijing Tiantan Hospital, Capital Medical University, Beijing 100070, China; rainietien@mail.ccmu.edu.cn (Y.T.); mxrdf0909@163.com (M.Z.); cyeeyeee@163.com (Y.C.); emmayang11111@outlook.com (M.Y.); 2 Chinese Institute for Brain Research, Beijing 102206, China; 3 China National Clinical Research Center for Neurological Diseases, Beijing 100070, China; 4 Advanced Innovation Center for Human Brain Protection, Capital Medical University, Beijing 100070, China; 5 National Center for Neurological Diseases, Beijing 100070, China

In the published publication [1], there was a mistake in the legend for Figure 2. The legend of Figure 2 was wrongly consistent with that of Figure 1. The correct legend appears below. The authors state that the scientific conclusions are unaffected. This correction was approved by the Academic Editor. The original publication has also been updated.

**Figure 2:** A brief overview of the glymphatic system. The glymphatic system consisted of three main components: cerebrospinal fluid (CSF) influx along periarterial spaces, exchange between CSF and interstitial fluid (ISF) in the brain parenchyma, and ISF efflux along perivenous spaces. Aquaporin4 (AQP4) located in astrocyte endfeet toward perivascular spaces were essential to maintain the normal function of the glymphatic transport, such as metabolic waste clearance. The loss of AQP4 polarization resulted in glymphatic failure in aging.
